# FOXP2 Expression and Oral Feeding Success in Preterm Infants: Sex 2 Differences

**DOI:** 10.3390/genes16020190

**Published:** 2025-02-04

**Authors:** Leonardo Henrique Ferreira Gomes, Andressa Brito Marques, Isabel Cristina de Meireles Dias, Daniela Prado Cunha, Hellen Porto Pimenta, Letícia da Cunha Guida, Sabrina Lopes Lucena, Adriana Duarte Rocha

**Affiliations:** 1 Laboratório de Alta Complexidade, Unidade de Pesquisa Clínica, Instituto Nacional da Saúde da Mulher, da Criança e do Adolescente Fernandes Figueira–Fundação Oswaldo Cruz, Rio de Janeiro 22250-020, Brazil; danielapradocunha@gmail.com (D.P.C.); leticia.guida@fiocruz.br (L.d.C.G.); 2 Unidade de Pesquisa Clínica, Instituto Nacional da Saúde da Mulher, da Criança e do Adolescente Fernandes Figueira–Fundação Oswaldo Cruz, Rio de Janeiro 22250-020, Brazil; andressacbmarques@yahoo.com.br (A.B.M.); fgisabelcmdias@gmail.com (I.C.d.M.D.); hellen.pimenta@fiocruz.br (H.P.P.); leohfg@gmail.com (S.L.L.)

**Keywords:** *FOXP2*, preterm infants, salivary samples, oral feeding

## Abstract

Background: The *FOXP2* gene, crucial for speech and motor functions, exhibits sex-specific expression differences. In premature infants, elevated *FOXP2* expression, particularly in females, correlates with improved oral feeding readiness, indicating the potential for enhancing neonatal care. Objective: This study investigates *FOXP2* gene expression in premature newborns across five feeding stages using salivary RNA, focusing on sex differences and their impact on oral feeding readiness to refine neonatal clinical protocols. Methods: *FOXP2* expression was analyzed using RT-qPCR and the ΔΔCt method across five feeding stages in 45 premature newborns using saliva-derived RNA (*n* = 225). Results: *FOXP2* expression increased significantly through feeding stages, especially in full oral feeding. Female infants showed consistently higher expression levels than males, with 58% higher expression by stage 5. Significant sex differences were apparent from stage 2. Conclusions: *FOXP2* expression impacts neuromuscular coordination and feeding readiness in preterm infants. The sex differences suggest that *FOXP2* could serve as a non-invasive biomarker for predicting oral feeding readiness, potentially improving clinical outcomes. Perspectives: *FOXP2* gene expression correlates with better oral feeding readiness in premature infants and may serve as a non-invasive biomarker to improve neonatal care. The study could enhance neonatal care, leading to improved outcomes and reduced hospital stays for preterm infants.

## 1. Introduction

The Forkhead Box Protein P2 gene (*FOXP2*) gene, located on chromosome 7, is crucial for the development of speech and language in humans, as initially discovered through studies of a family with a hereditary language disorder [1]. This transcription factor regulates the functions of multiple genes associated with neural development and synaptic function, and its role extends beyond language, influencing motor and behavioral development [2]. Recent research has highlighted sex-specific differences in *FOXP2* expression, which may contribute to various neurobiological and behavioral outcomes [3]. In the context of preterm infants, particularly regarding the development of oral feeding skills, *FOXP2* has gained attention for its potential role in feeding readiness. Oral feeding readiness involves the complex coordination of neuromuscular reflexes and feeding behaviors essential for neonatal survival and development. It has been observed that premature female infants often show a greater readiness for oral feeding than their male counterparts. These differences could be partially attributed to variations in *FOXP2* expression, impacting neuromuscular development related to sucking and swallowing [4,5].

Zimmerman et al. (2016) found significant correlations between salivary *FOXP2* expression and the success of oral feeding in preterm infants, suggesting a pivotal role of this gene in feeding readiness. This study aligns with the hypothesis that *FOXP2*’s involvement in motor coordination extends to feeding behaviors, underpinned by its expression in brain regions that are associated with motor control, such as the basal ganglia and cerebellum [4].

Gomes et al. (2024) validated the gene expression patterns in neonatal salivary samples, highlighting *FOXP2* as a crucial gene for oral feeding readiness. The transcription of genes such as *FOXP2* has been associated with the development of essential oromotor skills that are required for successful oral feeding, suggesting that the differential expression of these genes may indicate feeding readiness in premature infants [6].

The shared oromotor skills required for successful feeding and speech suggest that *FOXP2*’s influence is not limited to speech development but extends to essential feeding processes in neonates [7]. This is particularly relevant in preterm infants, who must develop adequate oral feeding skills before hospital discharge. Successful oral feeding necessitates the development and coordination of the nervous system, sensory systems, and muscular and digestive systems [8]. The challenge is compounded in preterm infants due to the need for rapid neuromuscular development and coordination. Clinical practices in neonatal intensive care units (NICUs) often rely on the subjective interpretation of physiological signs to initiate oral feeding. However, there is no standardized protocol, and decisions are frequently based on gestational age and weight rather than a comprehensive assessment of the infant’s sucking ability. Understanding the role of *FOXP2* in these processes could lead to better clinical protocols and interventions, potentially improving outcomes for premature infants [9].

This research aims to build upon the data published by our group [6], focusing on the expression of *FOXP2* in the saliva of premature infants, with a particular emphasis on sex differences observed at each stage of feeding: (1) no feeds, (2) partial enteral feeding, (3) full enteral feeding, (4) partial oral feeding, and (5) full oral feeding. By examining these differences, especially their impact on unsuccessful oral feeding, we seek to deepen our understanding of the molecular basis for sex differences in early development. These insights could significantly enhance clinical practices in neonatology. By further elucidating the role of *FOXP2*, we aim to improve the strategies that support the development of feeding skills in premature infants, ultimately leading to better health outcomes and reduced hospital stays.

## 2. Materials and Methods

### 2.1. Saliva Retrieval

In this study, the materials used were derived from Gomes et al., 2024 [6]. Saliva samples were prospectively obtained from 45 preterm infants (gestational age below 34 weeks) across five predefined feeding stages, resulting in a total of 225 samples. These stages were categorized as: (1) no feeds, (2) partial enteral feeding, (3) full enteral feeding, (4) partial oral feeding, and (5) full oral feeding. The saliva collection procedure adhered to established protocols [10]. Specifically, saliva was obtained using a 1 mL syringe connected to low-wall suction, minimizing the handling of the newborns, in compliance with hospital guidelines. To integrate with routine care and reduce stress, saliva collection was synchronized with other activities, such as diaper changes, when infants were more likely to cry and produce saliva.

The oropharyngeal area of the preterm infants was carefully aspirated, and the saliva was immediately preserved using 500 µL of RNA Protect Saliva (QIAGEN, Germantown, MD, USA), a solution designed to prevent changes in gene expression, inhibit microbial growth, and deactivate RNases. At each time point, two samples were gathered and stored at −80 °C until additional processing.

Only newborns without asphyxia were included in the sample, with asphyxia defined according to the guidelines set forth by the American Academy of Pediatrics (AAP) [11] and the American College of Obstetricians and Gynecologists (ACOG) [12]. The diagnosis of asphyxia required meeting all the following criteria: (i) severe metabolic or mixed acidosis (pH < 7.00) in an umbilical artery blood sample, if available, and (ii) a persistent Apgar score of 0–3 for more than 5 min.

The institution where the research was carried out has a human milk bank, so all preterm infants up to stage 4 use human milk (raw or from a human milk bank). 

The research was carried out in accordance with the Declaration of Helsinki and received approval from the Institutional Review Board of the Fernandes Figueira Institute (CAAE: 118 45767015.0.0000.5269). Informed consent was secured from all guardians of the participants involved in the study. Additionally, written consent was obtained from the guardians for the publication of this document.

### 2.2. RNA Isolation

RNA extraction from the saliva samples of preterm infants was performed using a modified TRIzol protocol (Thermo Fisher Scientific, Waltham, MA, USA), as outlined by Ghandi et al. (2020) [13]. The frozen saliva samples were thawed at room temperature without employing any rapid thawing methods to prevent RNA degradation. Approximately 1 mL of each sample was transferred into sterile DNase- and RNase-free 1.7 mL microcentrifuge tubes (Eppendorf, Hamburg, Germany) and centrifuged at 16,100 RCF for 20 min at 4 °C. The salivary supernatant was discarded, and each pellet was resuspended in 1 mL of TRIzol reagent (Thermo Fisher Scientific, Waltham, MA, USA) by pipetting and vortexing for 20 s to achieve homogenization, followed by a 5 min incubation at room temperature. Next, 200 μL of chloroform (Sigma-Aldrich, St. Louis, MO, USA) was added, the mixture was vortexed for 20 s, and then incubated at room temperature for an additional 5 min. The samples were centrifuged at 16,100 RCF for 20 min at 4 °C, and approximately 700 μL of the top aqueous layer was gently moved to fresh 1.7 mL microcentrifuge tubes free of DNase and RNase (Eppendorf, Hamburg, Germany). This chloroform extraction step process was repeated two more times, transferring smaller volumes of the aqueous phase: about 600 μL during the first repetition and 450–500 μL during the second. Each tube received 500 μL of cold isopropyl alcohol (Sigma-Aldrich), followed by 10 s of vortexing. The tubes were then incubated at −20 °C for at least one hour to precipitate the RNA. After incubation, the samples were centrifuged at 16,100 RCF for 20 min at 4 °C. The supernatant was carefully removed, and the pellet was washed with 1 mL of cold 80% molecular-grade ethanol, followed by centrifugation at 16,100 RCF for 5 min at 4 °C. This washing procedure was performed one more time. Excess ethanol was eliminated by pipetting after a brief centrifugation. The pellet was air-dried at room temperature for a minimum of 5 min, then resuspended in 20 μL of DNase- and RNase-free water and incubated in a 55 °C water bath for 5 min. After a brief vortex and a quick spin to collect the contents at the bottom of the tube, the RNA samples were stored at −80 °C.

The expression of the Forkhead Box Protein P2 (*FOXP2*) gene was subsequently assessed in these samples, with glyceraldehyde-3-phosphate dehydrogenase (*GAPDH*) and ribosomal 18S (*18S*) used as the internal control genes.

### 2.3. RNA Measurement

The RNA concentration (ng/μL) was determined using a Qubit 2.0 Fluorometer with the RNA Quantification Broad Range Assay Kit, as well as a NanoDrop 2000 Spectrophotometer (Thermo Fisher Scientific, Wilmington, NC, USA). Additionally, the RNA purity was assessed by measuring the 260/280 and 260/230 ratios using the NanoDrop 2000 Spectrophotometer. 

### 2.4. Quantitative Reverse Transcription PCR (RT-qPCR)

The expression levels of the *FOXP2*, *18S*, and *GAPDH* genes were evaluated at each collection stage through the quantitative reverse transcription PCR (RT-qPCR). The specific primers used are provided in the Supplementary Materials File S1. For cDNA synthesis, reverse transcription was performed with the SuperScript III First-Strand Synthesis System for RT-qPCR (Invitrogen, Waltham, MA, USA), utilizing 500 ng of RNA and a mixture of 2 pmol of each gene-specific primer, following the manufacturer’s guidelines. RT-qPCR was conducted on a 7500 Fast Real-Time PCR system (Applied Biosystems, Foster City, CA, USA) using Power SYBR Green PCR Master Mix (Applied Biosystems, Foster City, CA, USA), in accordance with the manufacturer’s instructions. Each reaction contained 1 μL of cDNA, 12.5 μL of 2× Power SYBR Green PCR Master Mix, and 1 μL of each primer (at a final concentration of 200 nM), for a total volume of 25 μL. The amplification protocol included an initial denaturation step at 95 °C for 10 min, followed by 40 cycles of 95 °C for 15 s and 60 °C for 1 min. The efficiency of the PCR and correlation coefficients were assessed using cDNA dilution curves, showing an efficiency greater than 92% and a correlation coefficient of ≥0.99 across all assays. To confirm the absence of genomic DNA and detect any primer dimers or contamination, each primer set included a reverse transcription negative control (without reverse transcriptase) and a non-template negative control. A melting curve analysis was performed to verify the specificity of the amplified products [6].

Relative mRNA expression was normalized against GAPDH and 18S mRNA, serving as internal reference genes, and the 2^−ΔΔCt^ method was applied for relative gene expression quantification [14,15]. Feeding stage 1 (no diet) was used as a control condition. The geometric mean of the Ct values for both reference genes was used to calculate ΔCts, with the equation: ΔCt = (Mean Ct FoxP2 of interest − ΔCt = (Mean Ct gene of interest − $\sqrt{\left(Ct\;GAPDH\times Ct\;18S\right)}$ [4,6]. All data were reported as the mean ± SD for each stage of the infants’ samples, with each sample analyzed in triplicate (raw data and analyses available in Supplementary Materials File S2.

### 2.5. Statistical Analysis

Statistical significance was set at *p* < 0.001 (**), and all statistical analyses were carried out using GraphPad Prism 5.0. (GraphPad Software Inc., San Diego, CA, USA). A one-way ANOVA with Greenhouse–Geisser correction was applied (refer to Supplementary Materials, File S3). For continuous variables, the Kruskal–Wallis test was employed.

## 3. Results and Discussion

The Fernandes Figueira National Institute for Women’s, Children’s, and Adolescents’ Health (IFF/Fiocruz) is home to the Human Milk Bank, which serves as the reference center for the Brazilian Network of Human Milk Banks (rBLH-BR), as well as for the Global Network. It is responsible for strategic actions in the sector, both within the scope of the Unified Health System (SUS) and International Technical Cooperation in Human Milk Banks. Thus, all preterm newborns up to stage 4 use human milk (raw or from a human milk bank). Other parameters considered were the gestational age at birth (an important determining factor: 32 vs. 34 weeks), oxygen requirement in hours, time of orotracheal tube use (h), and gestational age at stages 1–5. These parameters were analyzed, and no significant differences were observed between males and females (Table 1).

**Figure 1:**
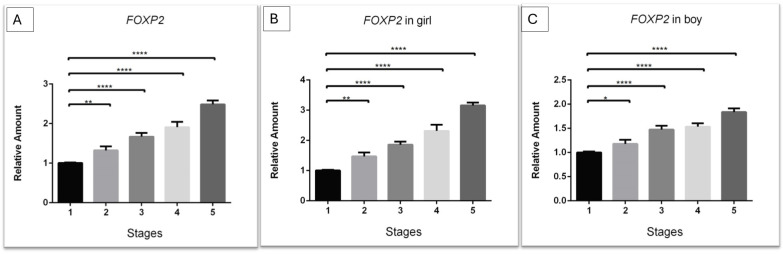
*FOXP2* gene expression across different feeding stages. Analysis includes all participants (**A**), females only (**B**), and males only (**C**). The data illustrate the fold change in relative expression through different stages relative to the control condition (stage 1, no feeds), using the qRT-PCR technique. The numbers 1–5 represent the five designated feeding stages at which newborn samples were gathered: (1) no feeds; (2) partial enteral feeding; (3) full enteral feeding; (4) partial oral feeding; and (5) full oral feeding. One-way ANOVA was used for statistical analysis. Error bars represent the standard deviation, and (*) denotes the level of significance (* *p* < 0.05; ** *p* < 0.01; **** *p* < 0.0001).

The gene expression profiles at various feeding stages were analyzed using the ΔΔCt method, with the results presented as relative expression changes (fold changes) (Figure 1). The expression of the *FOXP2* gene exhibited a trend of incremental elevation, beginning at stage 2 (partial enteral feeding) and nearly tripling by stage 5 (full oral feeding) when compared to the control group, stage 1 (no feeds) (Figure 1A). Upon examining the differential expression patterns between sexes, a notably more pronounced increase in gene expression was observed in female infants (1.47-fold, *** *p* < 0.001) compared to male infants (1.2-fold, ** *p* < 0.01) as early as stage 2. A closer inspection of the sex-specific graphs reveals that, generally, female infants consistently display a higher expression pattern than their male counterparts, as if they were “stages ahead” in terms of expression levels (stage 3: ♂ 1.47-fold vs. ♀ 1.85-fold; stage 4: ♂ 1.52-fold vs. ♀ 2.3-fold; and stage 5: ♂ 1.83-fold vs. ♀ 3.185-fold) (Figure 1B,C).

**Figure 2:**
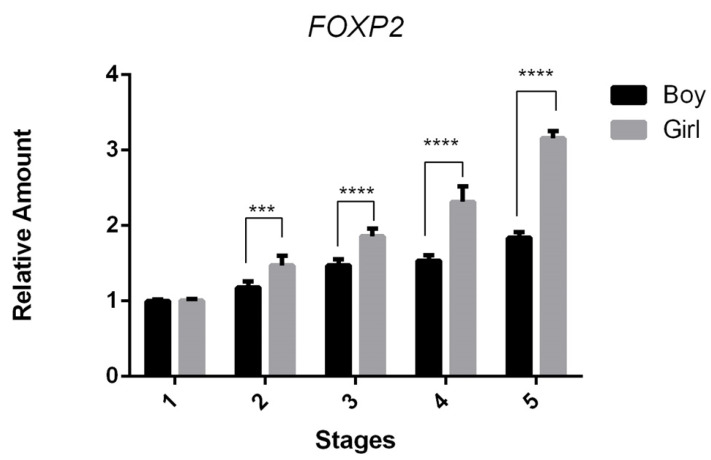
Comparison of *FOXP2* gene expression between male and female newborns across various feeding stages. The data illustrate the fold change in relative expression through different stages relative to the control condition (stage 1: no feeding), utilizing the qRT-PCR method. The numbers 1–5 represent the five designated feeding stages at which newborn samples were gathered: (1) no feeding; (2) partial enteral feeding; (3) full enteral feeding; (4) partial oral feeding; and (5) full oral feeding. Statistical analysis was conducted using a one-way ANOVA. Error bars indicate standard deviation, and (*) marks the significance levels (*** *p* < 0.001; **** *p* < 0.0001).

When directly comparing the values between male and female infants across different feeding stages, a significant sex difference is already detectable at stage 2 (♂ 1.2-fold vs. ♀ 1.47-fold, *** *p* < 0.001) (Figure 2). By the fifth stage (full oral feeding), this difference becomes even more pronounced, with female infants exhibiting a 58% higher expression compared to males (♂ 1.83-fold vs. ♀ 3.15-fold, **** *p* < 0.0001) (Figure 2).

Our research group has been actively investigating the issue of oral feeding readiness (Gomes et al., 2024) [6]. We validated gene expression patterns in neonatal salivary samples, where an increase in *FOXP2* gene expression was observed. In the current study, we also observed a similar pattern to that reported by Zimmerman et al. (2016) [4], with males exhibiting lower levels of *FOXP2* expression (higher Ct values) compared to females. Compared to the previous work [4], this study expands the sample size (45 vs. 21), presents a more balanced sex distribution (♂ 23 ♀ 22 vs. ♂ 8 ♀ 13), and examines gene expression across five different feeding stages. 

The transcription of genes such as *FOXP2* has been associated with the development of oromotor skills that are essential for successful oral feeding, suggesting that the differential expression of these genes may indicate feeding readiness in preterm infants [15]. This is particularly relevant for preterm neonates, who need to develop adequate oral feeding skills before hospital discharge. Successful oral feeding requires the maturation and integration of the nervous, sensory, muscular, and digestive systems, with *FOXP2* potentially playing a crucial role in this developmental process. The challenge is exacerbated in preterm infants due to the need for rapid neuromuscular development and coordination [16]. Clinical practices in neonatal intensive care units (NICUs) often rely on subjective interpretations of physiological signs to initiate oral feeding [17,18]. However, there is no standardized protocol, and decisions are frequently based on their gestational age and weight rather than a comprehensive assessment of their sucking ability. Understanding the role of *FOXP2* in these processes could lead to improved clinical protocols and interventions, enhancing the outcomes for preterm infants. Moreover, the potential use of salivary *FOXP2* expression as a biomarker for oral feeding readiness represents a promising avenue for non-invasive assessments in clinical settings [4,6,19].

These insights may enhance our understanding of the molecular bases of sex differences in early development and refine clinical practices in neonatology. Our next step involves a proteomic study aimed at elucidating the functions of *FOXP2* and considering the use of proteins and RNA as biomarkers, exploring material derived from various biological fluids such as saliva, peripheral blood, and dried blood spots on filter paper. This approach could improve our methodology for supporting clinical decision making regarding the development of feeding skills in preterm infants, ultimately aiming for better health outcomes and reduced hospitalization times.

## 4. Conclusions

The expression of *FOXP2* may influence neuromuscular coordination and readiness for feeding in premature infants, with sex differences in the expression of this gene. These findings suggest that *FOXP2* expression in saliva could serve as a non-invasive biomarker to predict oral feeding readiness in neonates, offering a new perspective for clinical interventions and improving feeding outcomes in premature infants. Therefore, the conclusion of this study is that there are differences in *FOXP2* gene expression between male and female newborns, and these findings could serve as a non-invasive biomarker to predict oral feeding readiness in this population, considering the difference in maturation between sexes.

## Figures and Tables

**Table 1:** Comparison of clinical parameters and feeding methods of premature newborns by sex and gestational stage.

	Males				Females				*p* *
	Mean ± DP	Median	Min	Max	Mean ± DP	Median	Min	Max	
GA at birth (weeks)	30.2 ± 2.4	30.4	25.4	34.0	30.0 ± 2.2	29.4	26.0	33.0	0.69
Oxygen requirement in hours	601 ± 753	240	0	2376	479 ± 721	240	0	2520	0.67
Time of orotracheal tube use (h)	92 ± 248	0	0	1152	51 ± 178	0	0	672	0.14
GA stage 1	30.2 ± 2.4	30.4	25.4	34.0	30.0 ± 2.2	29.4	26.0	33.0	0.69
GA stage 2	30.3 ± 2.3	31.0	25.5	34.0	30.1 ± 2.1	30.0	26.0	33.0	0.60
GA stage 3	31.3 ± 2.0	32.0	26.6	34.0	31.1 ± 2.1	31.0	27.0	33.0	0.68
GA stage 4	35.5 ± 2.9	35.0	32.0	44.0	34.2± 2.8	35.0	32.0	38.0	0.26
GA stage 5	36.6 ± 2.6	35.6	34.0	44.0	36.2 ± 1.4	35.4	34.0	40.0	0.86
	Breastfeeding	Bottle feeding	Breastfeeding + bottle feeding		Breastfeeding	Bottle feeding	Breastfeeding + bottle feeding		
GA Stage 5	22.7%	31.8%	45.4%		13.3%	20%	66.6%		

GA: gestational age; * Kruskal–Wallis test.

## Data Availability

The datasets produced and analyzed in this study are not publicly accessible due to confidentiality and ethical considerations related to patient information. However, they can be requested from the corresponding author upon reasonable request.

## Supplementary Materials

The following supporting information can be downloaded at: https://www.mdpi.com/article/10.3390/genes16020190/s1, File S1: Sequence of primers used in the study; File S2: Cts and ΔΔCt method; File S3: ANOVA test.

## Abbreviations

*18S*	ribosomal 18S gene
cDNA	complementary DNA
*FOXP2*	*Forkhead Box Protein P2 gene*
GA	gestational age
*GAPDH*	glyceraldehyde-3-phosphate dehydrogenase gene
IFF	Fernandes Figueira National Institute for Women’s, Children’s, and Adolescents’ Health
BLH-BR	Brazilian Network of Human Milk Banks
SUS	Unified Health System (Sistema Único de Saúde in portuguese)
mRNA	messenger RNA
NICUs	neonatal intensive care units
qPCR	quantitative polymerase chain reaction
RT-qPCR	reverse transcription quantitative PCR
