# Supplementary material for: FOXP2 Expression and Oral Feeding Success in Preterm Infants: Sex 2 Differences
Source: Genes (Basel). 2025 Feb 4;16(2):190. doi: 10.3390/genes16020190 (PMC11855131; doi:10.3390/genes16020190)
Supplement: Supplementary file 1 [file genes-16-00190-s001.zip › File S1-sequence primers.pdf]

File S1

Sequence of primers used in the study

Gene	Primer (5'-3')		Accession No
FOXP2	Foward Sequence	TGGATGACCGAAGCACTGCTCA	NM_148898
	Reverse Sequence	TGGGAGATGGTTTGGGCTCTGA	
18S	Foward Sequence	GCAGAATCCACGCCAGTACAAG	NM_022551
	Reverse Sequence	GCTTGTTGTCCAGACCATTGGC	
GAPDH	Foward Sequence	GTCTCCTCTGACTTCAACAGCG	NM_001256799
	Reverse Sequence	ACCACCCTGTTGCTGTAGCCAA	
